# Right Atrial Cardiac Calcified Amorphous Tumors in Young Women: Two Case Reports and a Narrative Review of the Literature

**DOI:** 10.3390/jcdd13070312

**Published:** 2026-07-07

**Authors:** Antonino M. Grande, Alessia Alloni, Davide Imò, Stefano Ghio, Eloisa Arbustini, Paolo Aseni, Andrea M. D’Armini

**Affiliations:** 1 Department of Cardiac Surgery, IRCCS Fondazione Policlinico San Matteo, 27100 Pavia, Italy; a.alloni@smatteo.pv.it (A.A.); d.imo@smatteo.pv.it (D.I.); a.darmini@smatteo.pv.it (A.M.D.); 2 Department of Cardiology, IRCCS Fondazione Policlinico San Matteo, 27100 Pavia, Italy; 3 Centre for Inherited Cardiovascular Diseases and Transplant Research Area-IRCCS Fondazione Policlinico San Matteo, 27100 Pavia, Italy; e.arbustini@smatteo.pv.it; 4 Department of Emergency Medicine, ASST GOM, Niguarda Hospital, 20162 Milan, Italy

**Keywords:** calcified amorphous tumor (CAT), cardiac mass, echocardiography, multimodality imaging, end-stage renal disease (ESRD), embolism, cardiac surgery, cerebrovascular accident (CVA)

## Abstract

Background: Cardiac calcified amorphous tumours (CATs) are rare non-neoplastic intracardiac masses characterized by calcified nodules within an amorphous fibrinous matrix and may clinically mimic thrombi or cardiac neoplasms. We report two uncommon cases of right atrial CAT occurring in young women and provide a narrative review of the literature. Methods: Two patients with right atrial CAT underwent multimodality imaging evaluation, including echocardiography, computed tomography, and cardiac magnetic resonance, followed by surgical excision and histopathological examination. A narrative review of published cases identified through PubMed and Embase between 1972 and 2025 was also performed. Results: The first patient presented with a calcified right atrial mass extending into the superior vena cava, associated with superior vena cava syndrome and autoimmune disease. The second patient, affected by end-stage renal disease on hemodialysis and thrombophilia, presented with a large calcified right atrial mass associated with a retained dialysis catheter fragment. Histopathological examination confirmed CAT in both cases. The literature review identified 112 published reports comprising 143 patients, including the two cases presented herein, highlighting frequent associations with end-stage renal disease, mitral annular calcification, and embolic complications. Conclusions: Cardiac CAT remains a rare and likely underrecognized entity with heterogeneous clinical presentation and significant embolic potential. Multimodality imaging is essential for diagnosis and surgical planning, while early surgical excision should be considered in symptomatic or high-risk patients.

## 1. Introduction

Cardiac calcified amorphous tumors (CATs) are extremely rare non-neoplastic intracardiac masses characterized histologically by nodular calcium deposits embedded within an amorphous fibrinous background. On echocardiographic evaluation, CAT has been reported in approximately 0.64% of patients with mitral annular calcification (MAC) and in 0.068% of the general population [1]. Despite increasing recognition, only a limited number of cases have been described in the literature, and optimal management of CAT remains incompletely understood, although increasing evidence suggests a potential role for chronic endothelial injury, thrombosis, and abnormal calcium-phosphate metabolism.

CAT may clinically mimic other cardiac masses, including myxomas, thrombi, infective vegetations, and primary or metastatic cardiac tumours, often posing a significant diagnostic challenge. Multimodality imaging therefore plays a pivotal role in the preoperative assessment and differential diagnosis of these lesions.

In this article, we report two uncommon cases of right atrial CAT occurring in two young women and describe their multimodality imaging findings, surgical management, and histopathological features. In addition, we performed a narrative review of CAT cases published between 1972 and 2025, including 143 patients (60 males and 83 females; age range: 15 days–89 years), with the aim of better characterizing the demographic distribution, anatomical localization, clinical presentation, imaging features, therapeutic strategies, and outcomes associated with this rare entity. By integrating two original cases with the available literature, we aim to provide a comprehensive contemporary overview of CAT and to highlight clinical and imaging features that may facilitate earlier diagnosis and appropriate management [2,3,4,5,6,7].

## 2. Materials and Methods

This study was designed to describe two original cases of cardiac calcified amorphous tumour (CAT) observed at our institution and to perform a narrative review of the literature in order to summarize the main clinical, imaging, pathological, and therapeutic characteristics of this rare entity.

The two clinical cases were retrospectively reviewed using data obtained from medical records, multimodality imaging examinations, surgical reports, and histopathological analyses. Follow-up information was collected from outpatient clinical and echocardiographic evaluations.

In addition, a literature search was performed using the PubMed and Embase databases to identify published reports of cardiac CAT from January 1972 to January 2025.

This review was conducted as a narrative review intended to provide a comprehensive overview of the published literature on cardiac calcified amorphous tumors (CATs). Although a structured search strategy was applied using the PubMed and Embase databases, the study was not designed as a formal systematic review and did not adhere to the Preferred Reporting Items for Systematic Reviews and Meta-Analyses (PRISMA) guidelines. No protocol registration, formal risk-of-bias assessment, or quantitative meta-analysis was performed. The objective of the review was descriptive and educational, focusing on the clinical, imaging, pathological, and therapeutic characteristics reported in individual cases and case series.

The literature search was performed using free-text keywords and databases indexing terms. The following search terms were used alone or in combination: “cardiac calcified amorphous tumour”, “calcified amorphous tumor”, “heart”, “cardiac CAT”, and “intracardiac calcified mass”. Articles written in English and reporting clinical cases or case series of histologically confirmed cardiac CAT were included. Articles identified through database searches were screened for relevance and eligibility. Conference abstracts, duplicate publications, non-English articles without sufficient clinical information, review articles without original cases, and reports lacking histopathological confirmation of cardiac CAT were excluded.

For each publication [2,3,4,5,6,7,8,9,10,11,12,13,14,15,16,17,18,19,20,21,22,23,24,25,26,27,28,29,30,31,32,33,34,35,36,37,38,39,40,41,42,43,44,45,46,47,48,49,50,51,52,53,54,55,56,57,58,59,60,61,62,63,64,65,66,67,68,69,70,71,72,73,74,75,76,77,78,79,80,81,82,83,84,85,86,87,88,89,90,91,92,93,94,95,96,97,98,99,100,101,102,103,104,105,106,107,108,109,110,111,112,113], the following variables were collected and are summarized in comprehensive Table S1 (additional material): number of reported patients, age and sex, anatomical localization of the lesion, associated comorbidities, presenting symptoms, imaging findings, treatment strategy (surgical or conservative), histopathological diagnosis, recurrence, and outcome.

The review aimed to provide an updated overview of the epidemiological distribution, clinical presentation, imaging characteristics, management strategies, and outcomes of cardiac CAT, as well as to compare these findings with the two cases presented herein. In addition, a narrative literature review was conducted to place the two cases into a broader clinical context and to summarize the published evidence on cardiac CAT.

## 3. Results

The two institutional cases are presented first, followed by the main findings of the narrative review of the literature.

### Case Reports

**Figure 1:**
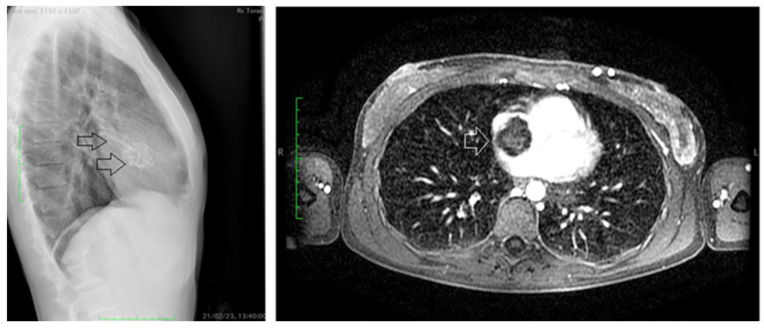
Case 1. Left panel: Right lateral chest radiograph showing the calcified amorphous tumour (arrows) extending from the superior vena cava into the right atrium. Right panel: Axial CT view demonstrating the calcified mass within the right atrium (arrow).

**Figure 2:**
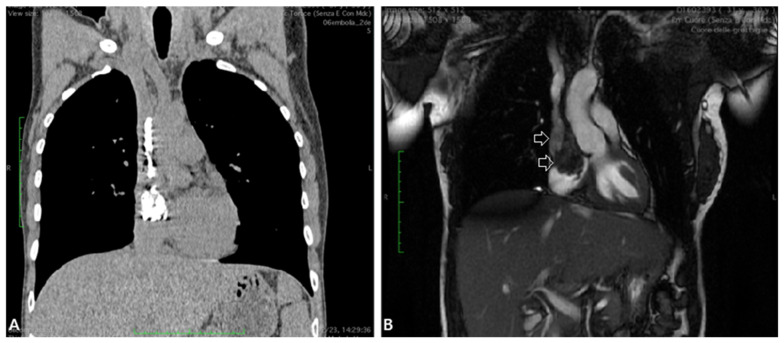
Case 1. (**A**) AngioCT, coronal view, showing the cardiac calcified amorphous tumor (CAT) in the superior vena cava and in the right atrium; (**B**) cardiac magnetic resonance image, coronal plane, showing the same lesion (arrows), with clear delineation of its intracardiac extent and relationship to adjacent structures.

Case 1. A 30-year-old woman was referred to our institution for surgical evaluation of a heavily calcified right atrial (RA) mass extending into the superior vena cava (SVC), causing clinical features consistent with SVC syndrome. The lesion had been incidentally detected during pre-pregnancy screening investigations after the identification of antiphospholipid antibodies and unexplained superficial venous collateral circulation over the left upper hemithorax. At admission, the patient reported progressive exertional dyspnea since late 2021, Raynaud phenomenon, recurrent digital ulcerations, and a history of mildly symptomatic SARS-CoV-2 infection in November 2021. Electrocardiography showed sinus rhythm. Chest radiography (Figure 1, left panel) demonstrated extensive intracardiac calcifications, while an Axial CT view demonstrated a calcified mass within the right atrium (right panel). Further evaluation previously performed at another institution included transesophageal echocardiography, Angio CT scan (Figure 2A, left panel) and cardiac magnetic resonance imaging (CMR) (Figure 2B, right panel), demonstrated a 4 cm calcified mass arising from the interatrial septum and extending into the SVC, causing partial obstruction of right ventricular filling and dilation with respiratory non-collapsibility of the inferior vena cava (IVC). Late gadolinium enhancement involving the inferior and inferolateral left ventricular walls suggested a previous ischemic event, likely secondary to coronary embolization. Coronary angiography excluded significant coronary artery disease.

At our center, contrast-enhanced computed tomography angiography (CTA) confirmed the presence of a calcified RA mass measuring 36 × 28 × 40 mm, extending approximately 6 cm into the SVC up to the azygos vein ostium (Figure 1 and Figure 2). CTA also demonstrated marked enlargement of the azygos and hemiazygos venous systems, multiple superficial collateral veins over the left hemithorax, congenital atresia of the left upper deep venous circulation, and occlusive lesions involving segmental and subsegmental pulmonary arterial branches of the left lower and middle lobes, consistent with previous pulmonary embolic events. Cardiac MRI findings were considered highly suggestive of calcified amorphous tumor (CAT). Given the complex anatomical findings, right heart catheterization was not performed, and surgical excision was indicated.

Surgery. Because of the unusual venous anatomy and SVC obstruction, cardiopulmonary bypass was established through peripheral femoral cannulation under mild sedation and spontaneous breathing before induction of general anesthesia. Median sternotomy was subsequently performed.

Systemic cooling to 24 °C induced ventricular fibrillation, and a left ventricular vent was inserted to optimize myocardial protection. Neither aortic cross-clamping nor cardioplegic arrest was required. Cerebral oxygen saturation was continuously monitored using near-infrared spectroscopy (NIRS).

**Figure 3:**
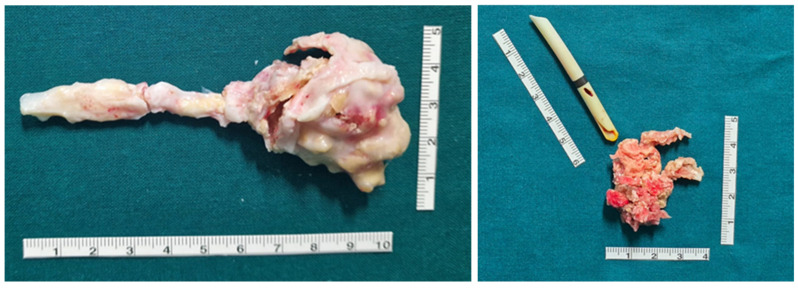
(**Left**) Case 1. The excised cardiac CAT; note the superior vena cava and right atrial mold. (**Right**) Case 2. The large, 4 cm diameter calcified mass was firmly adherent to the inferior vena cava inlet and extended toward the tricuspid valve. The mass was completely excised, along with a segment of a dialysis catheter in the superior vena cava, shown at the top.

Right atriotomy and longitudinal SVC incision extending to the brachiocephalic veins were performed. The calcified mass was firmly adherent to the interatrial septum above the fossa ovalis and close to the IVC ostium. Complete excision of the mass and its SVC extension (Figure 3) was achieved using alternating periods of circulatory arrest and reperfusion. Intraoperatively, complete occlusion of the left brachiocephalic vein and atresia of the left upper deep venous system were confirmed. The RA was closed, systemic temperature was restored to normothermia, and the heart resumed spontaneous sinus rhythm. The patient was successfully weaned from cardiopulmonary bypass without complications.

Postoperative Course and Histopathology. The postoperative course was uneventful. The patient was extubated on postoperative day 1 and discharged home on postoperative day 8 with an indication for long-term anticoagulation therapy using vitamin K antagonists.

Predischarge transthoracic echocardiography showed preserved biventricular function, mild apical hypokinesia, normal estimated pulmonary artery pressure, and residual increased echogenicity at the RA roof without evidence of residual obstruction.

Histopathological examination demonstrated extensively calcified, predominantly acellular material embedded within a fibroconnective capsule, with focal lymphocytic inflammatory infiltrates, confirming the diagnosis of cardiac calcified amorphous tumor. Follow-Up. At 2-year follow-up, the patient remained asymptomatic, and serial transthoracic echocardiography demonstrated no evidence of CAT recurrence. Rheumatologic reassessment established the diagnosis of systemic lupus erythematosus and antiphospholipid syndrome, supporting the indication for lifelong anticoagulation and multidisciplinary follow-up.

**Figure 4:**
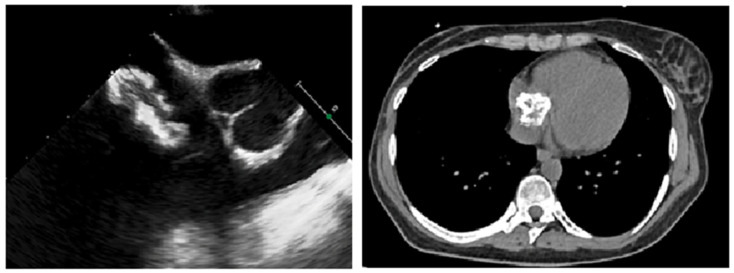
Case 2. (**Left**) TEE shows the calcified amorphous tumor (CAT) in the right atrium; (**Right**) axial contrast-enhanced CT angiography view showing the right atrial mass.

Case 2. A 31-year-old woman with end-stage renal disease (ESRD) on chronic hemodialysis was referred to our institution because of progressive dyspnea. Her medical history included hypertensive nephropathy, chronic anemia, hyperhomocysteinemia, and heterozygous G20210A prothrombin gene mutation. Transesophageal echocardiography (TEE) demonstrated a large heterogeneous right atrial mass measuring 34 × 25 mm, adherent to the lateral atrial wall near the inferior vena cava (IVC) inflow, which remained patent (Figure 4, left panel). Two additional highly mobile echogenic components measuring 12 × 7 mm and 10 × 6 mm, respectively, were also identified. Moderate tricuspid regurgitation was present. The IVC was dilated (22 mm) but showed preserved inspiratory collapse. Computed tomography angiography (CTA) confirmed the presence of a heavily calcified mass attached to the right atrial wall with extension toward the superior vena cava (Figure 4, right panel). Before referral for surgery, an attempt at endovascular thrombectomy using interventional radiology was unsuccessful as the thrombus was extensively calcified, making it resistant to endovascular removal. This aligns with findings by Yew and Leong [114], who noted that catheter-related thrombi, especially calcified ones, pose significant challenges to non-surgical management. Given the high embolic risk associated with the mobile components of the mass, surgical excision was indicated (Figure 3, right panel).

Postoperative Course and Follow-Up. The patient was successfully extubated in the early postoperative period. Because of volume overload, ultrafiltration dialysis was required postoperatively. Persistent fever developed despite repeatedly negative blood cultures, and empirical broad-spectrum antibiotic therapy with daptomycin and meropenem was initiated. Fever resolution was ultimately achieved after removal of the femoral dialysis catheter and placement of a new tunneled vascular access. Histopathological analysis confirmed the diagnosis of calcified amorphous tumor. At 12-month follow-up, the patient remained clinically stable, with no evidence of recurrence on transthoracic echocardiography, and continued regular hemodialysis treatment.

To place these two cases into a broader clinical context, we summarized the main findings of our narrative review of the published literature on histologically confirmed cardiac CAT. After excluding duplicate and non-eligible reports, the review included 112 published articles reporting a total of 143 patients with CAT between 1972 and 2025 [2,3,4,5,6,7,8,9,10,11,12,13,14,15,16,17,18,19,20,21,22,23,24,25,26,27,28,29,30,31,32,33,34,35,36,37,38,39,40,41,42,43,44,45,46,47,48,49,50,51,52,53,54,55,56,57,58,59,60,61,62,63,64,65,66,67,68,69,70,71,72,73,74,75,76,77,78,79,80,81,82,83,84,85,86,87,88,89,90,91,92,93,94,95,96,97,98,99,100,101,102,103,104,105,106,107,108,109,110,111,112,113] (Table 1 and Table S1).

Most articles described single-case reports, while only two reported larger case series. The largest cohorts were published by Reynolds et al. [3] (11 patients) and Yilmaz et al. [46] (12 patients). Recently, Streian [107] reported 16 cases published between 2020 and 2024.

Patients’ age ranged from 15 days to 89 years, with a slight female predominance. CAT most frequently involved left-sided cardiac structures (98 cases, 68.5%), particularly the mitral valve and annulus (54 cases, 37.76%) and left ventricle (20 cases, 13.9%), whereas right-sided localization was less common (25.9%). End-stage renal disease (ESRD), mitral annular calcification (MAC), diabetes mellitus, valvular heart disease, and hypercoagulable conditions represented the most frequently associated comorbidities (see Table 1).

Dyspnea was the most common presenting symptom, followed by cerebrovascular and embolic events, syncope, and heart failure manifestations. However, a considerable proportion of lesions were incidentally discovered during imaging examinations performed for unrelated clinical conditions.

Surgical excision represented the predominant treatment strategy in 123 patients (86%), allowing for definitive histopathological diagnosis in most cases, and was generally associated with favorable outcomes, with an operative mortality of 2.8%, although isolated cases of recurrence and postoperative mortality were reported. In the following sections, we describe in detail two additional cases of right atrial CAT observed at our institution, both occurring in young women and characterized by uncommon clinical and anatomical features.

## 4. Discussion

Cardiac calcified amorphous tumors (CATs) are rare non-neoplastic intracardiac masses characterized by calcified nodules embedded in an amorphous fibrinous matrix. Despite increasing recognition, their pathogenesis remains incompletely understood, and their clinical presentation is highly heterogeneous. CATs may be discovered incidentally or may present with symptoms related to intracardiac obstruction or systemic/pulmonary embolization. Because these lesions may mimic thrombi, myxomas, vegetations, or other cardiac tumors, diagnosis remains challenging and often requires multimodality assessment.

Although CATs were formally defined as a distinct histopathological entity by Reynolds et al. [3] in 1997, Fleming and Stovin [2] had already described a calcified right atrial mass in 1972.

Available evidence suggests that CAT development is multifactorial. End-stage renal disease, abnormalities of calcium-phosphate metabolism, hypercoagulability, chronic inflammation, and endothelial injury have all been implicated as potential contributing factors. In our two patients, these mechanisms appeared particularly relevant: the first patient had autoimmune and prothrombotic disease, whereas the second had ESRD on chronic hemodialysis, thrombophilia, and a retained dialysis catheter fragment. These findings further support the hypothesis that thrombotic predisposition and chronic endothelial injury may contribute to the formation of calcified intracardiac masses.

Our literature review confirms that CAT is more commonly observed in older patients and more frequently involves left-sided cardiac structures, especially the mitral valve/annulus and left ventricle. By contrast, both of our cases showed the uncommon pattern of right atrial involvement in young women, with extension to the superior or inferior vena cava. These observations broaden the anatomical and clinical spectrum of CATs and highlight the need to consider this diagnosis even in atypical right-sided lesions.

Embolic manifestations emerged as one of the major clinical complications in the reviewed cases, particularly in the presence of mobile lesions, including the so-called “swinging CAT” phenotype. Rapid lesion growth has also been described in selected cases, especially in association with mitral annular calcification. In this context, both of our patients presented features associated with increased embolic risk, supporting the importance of early recognition and close clinical assessment [115].

Imaging plays a central role in the diagnosis and management of CATs. Echocardiography is usually the first-line modality and is essential for identifying lesion mobility and hemodynamic consequences. Computed tomography is particularly helpful in defining the extent of calcification and extracardiac or venous extension, while cardiac magnetic resonance can provide additional anatomical and tissue characterization. As shown in our cases, integration of these imaging techniques is especially valuable for differential diagnosis and preoperative planning.

Surgical excision remains the treatment of choice in most symptomatic patients and in those with uncertain diagnosis or significant embolic risk. In our review, surgery was the predominant strategy and was generally associated with favorable outcomes, whereas recurrence was uncommon and has mainly been reported after incomplete resection. Our second case also illustrates the limitations of percutaneous treatment when the mass is heavily calcified, further supporting the role of surgery in selected high-risk patients.

This review highlights key issues surrounding this rare disease, which remains underreported due to limited awareness. Patients from East Asia accounted for nearly 40% of the entire cohort (57 patients). Furthermore, an analysis of the treating centers revealed that 54 of the 112 total reports (48.2%) originated from the Far East, specifically Japan (44), China (4), South Korea (4), Hong Kong (1), and Taiwan (1). Between 2018 and 2025, 61 reports were published, with 35 (57.4%) originating from Far Eastern centers—likely reflecting a stronger regional interest in the CAT topic [116].

Finally, the increasing number of published reports in recent years likely reflects greater awareness of this entity and wider use of multimodality imaging rather than a true rise in disease incidence. Nevertheless, CATs probably remain underrecognized because of their rarity and their overlap with other intracardiac masses.

## 5. Conclusions

A cardiac calcified amorphous tumor (CAT) is a rare and likely underrecognized non-neoplastic intracardiac mass with heterogeneous clinical presentation and significant obstructive or embolic potential. Our two cases broaden the clinical spectrum of CATs by showing uncommon right atrial involvement in young women with thrombotic and inflammatory backgrounds, supporting a possible role of chronic endothelial injury and thrombosis in their development. Because CATs may mimic thrombi, vegetations, or other cardiac tumors, multimodality imaging is essential for diagnosis and surgical planning, while surgical excision should be considered in symptomatic patients and in those with mobile or high-risk lesions; long-term follow-up may also be advisable in patients with persistent predisposing conditions.

## Figures and Tables

**Table 1:** Main findings of the literature review. Summary of the principal demographic, anatomical, clinical, and therapeutic findings derived from the review of 112 published studies of cardiac calcified amorphous tumor (CAT), with 143 cases reported between 1972 and 2025.

Variable	Findings
Total reported patients	143 patients
Male/female	60 (41.96%)/83 (58%)
Age range	15 days–89 years
Patients ≥ 50/≥60 years old	96 (67.1%)/80 (55.9%)
Patients from East Asia	57 (39.8%)
Left-sided localization	98 (68.5%)
Right-sided localization	37 (25.8%)
Both left- and right-sided localization	4 (2.8%)
Ascending aorta localization	4 (2.8%)
Left atrial involvement	13 (9.1%)
Left ventricular involvement	20 (13.9%)
Right atrial involvement	19 (13.3%)
Biatrial involvement	1 (0.7%)
Right ventricular involvement	15 (10.5%)
Mitral valve (MV) isolated involvement	40 (27.9%)
Mitral valve total involvement	54 (37.7%)
Aortic valve (AV) isolated involvement	4 (2.8%)
AV/MV	1 (0.7%)
AV/TV	1 (0.7%)
AV/LV	1 (0.7%)
Combined LA/MV	2 (1.4%)
Combined LA/MV/LV involvement	1 (0.7%)
Combined LA/MV/LV	9 (6.3%)
Combined MV/TV involvement	1 (0.7%)
End-stage renal disease/hemodialysis	44 (30.8%)
Mitral annular calcification (MAC)	25 (17.5%)
Dyspnea	45 (31.5%)
Cerebrovascular events	30 (20.1%)
Pulmonary embolism	7 (4.9%)
Syncope	16 (11.2%)
Heart failure	11 (7.7%)
Asymptomatic cases	34 (23.8%)
Diabetes mellitus	16 (11.2%)
Hypertension	24 (16.8%)
Coronary artery disease	12 (8.4%)
Surgical treatment	123 (86.%)
Medical treatment only	11 (7.7%)
Post-diagnosis deaths or unknown outcomes	8 (5.6%)
Catheter AngioVac	1 (0.7%)
Operative mortality	4 (2.8%)

## Data Availability

The data supporting the findings of this study are available within the article and its Supplementary Materials. Additional anonymized data are available from the corresponding author upon reasonable request, subject to privacy and ethical restrictions.

## Supplementary Materials

The following supporting information can be downloaded at: https://www.mdpi.com/article/10.3390/jcdd13070312/s1, Table S1. Clinical issues of cardiac CAT cases published from 1972 to 2025.

## Abbreviations

Abbreviations and acronyms used in Table 1 and Table S1 (Supplementary Materials).

AA = ascending aorta; AV = aortic valve; CAT = calcified amorphous tumor; ESRD = end-stage renal disease; LA = left atrium; LV = left ventricle; MV = mitral valve; RA = right atrium; RH = right heart; RV = right ventricle; TV = tricuspid valve; AC = aortic coarctation; AF = atrial fibrillation; AMI = acute myocardial infarction; AR = aortic regurgitation; ASD = atrial septal defect; Asymp = asymptomatic; AT = atrial tachycardia; AV = atrio-ventricular; AVR = aortic valve replacement; AVS = aortic valve stenosis; CAB = coronary artery bypass; CAD = coronary artery disease; CALC = calcified; CAT = calcified amorphous tumor; CVA = cerebrovascular accidents; CHT = chemotherapy; CI = cerebral infarction; CMS = calcified mass; COPD = chronic obstructive pulmonary disease; CRAO = central retinal artery occlusion; CRYO = cryoablation; CS = coronary sinus; CT = computed tomography; CXR = chest radiography; DM = diabetes mellitus; DYSA = dysarthria; DYSL = dyslipidemia; DYS = dyspnea; END = endocarditis; ENPH = emphysema; ESRD = end-stage renal disease; EV = eustachian valve; FO = foramen ovale; FU = follow-up; GP = gastric pain; HC = hepatic cirrhosis; HD = hemodialysis; HF = heart failure; HYL = hyperlipidemia; HYP = hypertension systemic; HPT = hyperparathyroidism; IAS = interatrial septum; IS = ischemic stroke; IVC = inferior vena cava; LA = left atrium; LM = left main coronary artery; LV = left ventricle; LVEF = left ventricle ejection fraction; LVOT = left ventricle outlet tract; MA = mitral annulus; MAC = mitral annulus calcification; MOF = multiorgan failure; MR = mitral regurgitation; MRI = magnetic resonance imaging; MS = membranous septum; MT = medical therapy; MTH = month; MTHAL = major thalassemia; MVR = mitral valve replacement; MYX = myxoma; PAA = pulmonary arteries; PD = peritoneal dialysis; PDA = patent ductus arteriosus; PE = pulmonary embolism; PEA = pulmonary endoarterectomy; PF = pulmonary fibrosis; PH = pulmonary hypertension; PLMV = posterior leaflet MV; PLP = palpitation; PM = papillary muscle; PMK = pace-maker; PT = pulmonary trunk; PV = pulmonary valve; PYE = pyelonephritis; RA = right atrium; RCA = right coronary artery; RT = radiotherapy; RV = right ventricle; SMK = smoker; STEMI = ST elevation myocardial infarct; STJ = sinotubular junction; SV = sinus of Valsalva; SVC = superior vena cava; SYN = syncope; TA = tricuspid annulus; TEE = transesophageal echocardiography; TIA = transient ischemic attack; TR = tricuspid regurgitation; TR = tricuspid regurgitation; TTE = transthoracic echocardiography; UK = unknown; VL = vision loss; VT = ventricular tachycardia; WKE = week; YR, YRS = year, years patients.
