# Supplementary material for: Right Atrial Cardiac Calcified Amorphous Tumors in Young Women: Two Case Reports and a Narrative Review of the Literature
Source: J Cardiovasc Dev Dis. 2026 Jul 7;13(7):312. doi: 10.3390/jcdd13070312 (PMC13411230; doi:10.3390/jcdd13070312)
Supplement: Supplementary file 1 [file jcdd-13-00312-s001.zip › jcdd-4394257-supplementary.pdf]

Author	N.°	Age/sex	Site	Comorbidities / Diagnosis	Symptoms	Treatment
Fleming [2] 1972 UK	1	40 / F	RA RV PT IVC	HF, HC, PYE, HYP. CXR: cardiac enlargement; cath: RA calc mass diagnosed as MYX.	DYS, leg edema 6 MTHs before, periorbital edema, hepatomegaly.	Surgery: excision RA mass calc. polypoid 7x5x5cm, IVC, RV/PT; TVR. Death 4th day.
Reynolds [3] 1997 U.S.A.	11	16 / M<br>30 / M<br>33 / F<br>34 / F<br>48 / F<br>60 / F<br>65 / M<br>67 / F<br>67 / M<br>73 / F<br>75 / F	LA<br>LV<br>RV<br>RV<br>MV<br>LV, MV<br>RV, TV<br>RA<br>LV<br>RA, SVC<br>LV	RT/chemotherapy 3 months<br>None<br>Recurrent PE<br>Systemic Lupus-like illness<br>MR, cleft MV, TR<br>MR, AR<br>CAD, recurrent PE<br>CAD, HF<br>ESRD, CAD, tumoral calcinosis<br>Diverticulitis, parenteral nutr. DM	Exertional DYS, near-SYN<br>Chest pain, palpitation, SYN<br>DYS<br>Vertigo, orthopnea<br>CVA<br>CVA, retinal emboli<br>DYS<br>SYN<br>SYN<br>Dizziness, exertional DYS<br>"Funny sensation in the chest"	Surgery<br>Surgery<br>Surgery<br>Surgery<br>Surgery<br>Surgery<br>Death 1 month after diagnosis<br>Surgery<br>Surgery<br>Surgery<br>Surgery
Chaowalit [4] 2005 U.S.A.	1	20 / F	RV	Chest trauma 3 ys earlier	PE, DYS	Surgery
Lewin [5] 2006 U.S.A	1	60 / F	RV	No	SYN	Surgery, died 1 day after
Fealey [6] 2007 U.S.A.	1	20 / F	RV	No. TTE	PE, DYS, fatigue	Surgery: incomplete resection, recurrence 28 months later
Khulbey [7] 2008 India	1	26 / M	RA	17 y earlier ASD closure	Fever, fatigue	Surgery
Inamdar [8] 2008 U.S.A.	1	85 / F	MA	MAC, HYP, ESRD, DM	Chronic fatigue	Surgery
Ho [9] 2008 U.S.A.	1	44 / M	MV/LV	CT: LV diffuse calcific infiltration PMs and chordae	Previously healthy, DYS	Surgery avoided for huge mass infiltration, referred for Heart Transplant
Gutierrez-Barros [10] 2008 Spain	1	35 / M	RA	HYP, Alport syndrome, rejected kidney transpl., ESRD	Septic shock. TTE/TEE: mass 33x23mm in RA at the EV near IVC.	Surgery: excision. Post-op. uneventful. FU 2 months: well.
Flynn [11] 2009 India	1	Young man	TV/RV and LPA	TTE: pedunculated mobile calc mass TV chordae.	SYN, PE	Surgery: excision and pulmonary TEA
Habib [12] 2010 U.S.A.	1	58 / F N	MV, LV infiltration	Pre-ablation intracardiac echo endomyocardial calcif. pattern	Cardiac arrest and recurrent ventricular tachycardia	MT
Gupta [13] 2010 India	1	40 / F	RA	No	DYS, fatigue	Surgery
Vaideeswar [14] 2010 India	2	56 / M<br>35 / M	RA IVC<br>RA IVC	NA<br>NA	DYS, PE, blurring vision<br>DYS, Dizziness walking, PE	Surgery: excision, pulmonary TEA; died 1 week after surgery
Kubota [15] 2010 Japan	2	64 / F<br>44 / M	MA<br>LV PM	ESRD, HD, DM<br>ESRD, HD, Lupus	Incidental TTE mass 3x27mm<br>Incidental, TTE mass 5x28mm	Surgery: excision and MVR/AVR<br>Surgery: excision, discovered 2nd in LV. FU: 3ys both well.
Greaney [16] 2011 UK	1	69 / F	LV MV	COPD, TTE: 2cm LVOT mass	DYS, LV failure, HF, stroke	Surgery
Ananthakrishna [17] 2011 India	1	45 / F	LV	Rheumatic heart disease. TTE: significant MV and AV disease	DYS	Surgery
Vlasseros [18] 2011 Greece	1	65 / F N	LV MV	DM, HYP	Retinal arterial emboli and transient visual field defects	Surgery
Lin [19] 2011 Taiwan	1	74 / F A	LA	ASYM. TTE: 14x27 mass attached to IAS	Incidental: chest X-ray calcifications cardiac silhouette	Surgery: excision.<br>FU: 6 MTHs doing well.
De Sousa [20] 2011 Brasill	1	17 / M	TV	Ebstein anomaly. TTE: calcified mass TV post. leaflet 22x14 mm	Cardiomegaly, ASYM	Surgery: excision, TV plasty
Hyun [21] 2011 Korea	1	33 / M A	RV	No<br>CT: PE, calcified mass in RV.	Inferior limbs edema, DYS. PE.<br>MRI: calc mass from TV to RVOT	MT: anticoag. Considered for Heart-Lung transplant.
Fujiwara [22] 2012 Japan	2	58 / M<br><br>65 / M	MA<br><br>MA	ESRD, MAC.<br><br>ESRD, MAC. TTE: mobile mass ventricular side of MAC	Incidental. TTE 2 tumors: 1 on atrial and the second on LV side.<br>Incidental.	Surgery<br><br>Surgery
Nishigawa [23] 2012 Japan	1	78 / F	LA	MAC	Incidental	Surgery
Nazli [24] 2013 Turkey	1	54 / F	LV	Hypothyroidism	CRAO	Surgery
Kawata [25] 2013 Japan	1	59 / M	MA	ESRD, DM, MAC. TTE/TEE: high-echoic mobile tumor (6×28 mm)	Incidental	Surgery
Rehman [26] 2014 U.S.A.	1	72 / F	RV	ASD. TEE: ASD with RV 3×2cm mobile mass from apex	DYS for 2 months, PE	Surgery
Mohamedali [27] 2014 U.S.A.	1	69 / F	MA	ESRD, MAC	DYS, epigastric pain	Surgery
Yamamoto [28] 2014 Japan	1	82 / F	MA	NK, MAC. TTE: entrapment of the mass in a stenotic AV	Progressive HF.	Surgery
Choi [29] 2014 U.S.A.	1	57 / F	RA	Multiple medical problems.<br>TTE: 2 x 1.7 cm RA mass	DYS, fever	Surgery: excision.<br>FU 1year free of disease.
Hussain [30] 2014 U.S.A.	3	80 / F<br>69 / F<br>60 / F	LV<br>MV<br>RA	CAD, HYP.<br>Lung adenocarcinoma<br>Breast cancers 10 yrs earlier	SYN<br>PLP<br>DYS	Surgery<br>Surgery MV plasty<br>Surgery

Suh [31] 2014 Korea	1	70 / F	LA	DM, HYP. TTE/TEE LA IAS calcif. mass 2cm above FO	DYSA, right side weakness. MRI: CI	Surgery
Sabzi [32] 2014 Iran	1	77/ M	RA	No	DYS, asthenia, cyanosis. TEE: 4x4 cm calcified mass at FO.	Surgery: Excision
Tanaka [33] 2015 Japan	1	66 / F	LA	PD (11 years), DM	Incidental, TTE 4 yrs before: mobile MV mass attached, 1cm.	Surgery: excision.<br>Post-op was uneventful.
Yasui [34] 2015 Japan	1	67 / F	LV	No	DYS 10 yrs. TTE: mobile mass 10mm	Surgery: excision. 2 yrs later recurrence in LV, EF 0.40.
Masuda [35] 2015 Japan	1	69 / F	MV	Pulm. tuberc., HYP 3 yrs earlier TTE: calcif. mass PLMV.	Incidental. TTE showed a 19x18 mm mass between P2 and P3.	Surgery: excision, PLMV pericardial patch.
Kinoshita [36] 2015 Japan	1	70 / F	MA TA	HD 23 yrs; 2 yrs earlier PMK. TEE masses MA/septal TA.	Exertional DYS, lower limb edema, acute HF.	Surgery: MVR, TVP, excision; PM leads extract for suspected END.
Prifti [37] 2015 Albania	1	32 / M	RV	No. CT: mass 4x10cm in RV, RPA occlusion.	Edema, cough, DYS. TTE/TEE: calc RV mass from TV to PV/PT.	Surgery: excision. TRPA, PVR, patch PT. FU: 1 year doing well.
Katsuki [38] 2016 Japan	1	85 / F	MA	MAC	Right brain stem infarction	Surgery
Guedes [39] 2016 Portugal	1	79 / F	MA	Coronary angiogram revealed left main and 3-vessel disease.	Angina. Incidentally, TTE: MV mobile mass protrud into LVOT	Surgery: CAB and mass excision
Takeuchi [40] 2016 Japan	1	60 / M	MA	HD. TTE routine: the mass developed in 2-year FU.	Incidental, ASYM	Surgery: MVR and excision
Seo [41] 2016 Japan	1	72 F A	MV / LVOT	No	DYS. TTE: mobile mass MV subvalvular extending to LVOT.	Surgery: excision through AV, from PM / chordae tendineae.
Watanabe [42] 2016 Japan	1	81 M	AA SV	ESRD	HF for severe AS. TEE: SV mass 15.3x9.9 mm	Surgery: excision
Padang [43] 2016 U.S.A.	1	59 / M	IVC, RA, RV	CAT surrounding a broken line	ASYM	Surgery
Miyano [44] 2016 Japan	1	49 / F	MA / LA	HD for 6 yrs. mild MVR and severe AS.	TTE for AS, mass in posterior MA with rapid growth to 25×27mm.	Surgery: AVR, MA decalcified using Cavitron Aspirator; 3 wks later surgery for LA mass.
Zairi [45] 2016 Tunis	1	5 / F	RA / RV, total 4 masses	No	SYN. TTE: non-mobile mass in RA attached to IAS.<br>FU 4ys doing well.	Surgery: RA: 1st mass 2x1x1cm on FO, 2nd mass on IVC outlet; RV: 1st mass 4x3x2 mm behind ALTV, 2nd near PA.
Yilmaz [46] 2016 Turkey	12	60 / M<br>78 / F<br><br>74 / F<br>50 / F<br><br>66 / F<br><br>51 / F<br><br>81 / F<br><br>57 / F<br><br>85 / F<br><br>27 / M<br><br>87 / F<br>68 / M	LV, AV<br>LV<br><br>LV, MV<br>LV, PM<br><br>LV<br><br>LV, MV<br><br>LV<br><br>LV, MV<br><br>LV<br><br>LA, PM<br><br>LV<br>LV	CT, MRI mass 35x15 mm<br>Chest X-ray, MRI 40x32 mm calc; CT, MRI<br>27x21 mm polyp.<br>CT 13x10 mm polypoid calcified<br>Chest X-ray, MRI 20x16 mm calc<br>ESRD; CT, MRI, 17x14 mm calc<br>CT, MRI, 33x16 mm calc. mass<br>ESRD; CT, MRI, 15x17mm polyp<br>CT, 43x28 mm infiltrative, calcif<br>ESRD; CT 20x11 infiltrative, calc CT, 37x14 mm ovoid calc. mass<br>CT, 40x10 tubular, infiltrat calc	SYN<br>ASYM<br><br>ASYM, ESRD<br>ASYM<br><br>DYS<br><br>DYS<br><br>ASYM<br><br>DYS<br><br>ASYM<br><br>CRAO<br><br>ASYM<br>ASYM	Surgery: Excision<br>NK<br><br>Surgery: Excision<br>NK<br><br>Surgery: Excision<br><br>Surgery: Excision<br><br>NK<br><br>Surgery: Excision<br><br>Surgery: Excision<br><br>Surgery: Excision<br><br>NK<br>NK
Kyaw [47] 2017 U.S.A.	1	68 / F	MV	40-pack-year smoking history but no known medical problem	STEMI and occipital stroke	Surgery
Nakamaru [48] 2017 Japan	1	70 / M	MV	ESRD, MAC. CRX: cardiomegaly; TTE MAC / mass 8×6 mm PLMV	Chest discomfort during exercise	Surgery
Bonta [49[2017 The Netherlands	1	42/ M	PT, RV	Not known	Exertional DYS. TTE: PT obstruct. by calc mass, RV mass, TR, PH.	Surgery: excision through PEA, TVR.
Yoshimura [50] 2017 Japan	1	64 / F	MV	ESRD, HD for 20 yrs, DM, CAD stent 12 yrs earlier, fem. fract.	Incidental. TTE / TEE, not seen 3 yrs earlier, 15 mm mass MV.	Surgery
Tao [51] 2017 Japan	1	61 / M	LVOT	ESRD for 10 yrs, previous AMI, AF, TTE, TEE	ASYM	Surgery: ACB, Maze, excision
Chowdhary [52] 2017 India	1	73 / M	RA LA	Inveterate smoker.	Exertional DYS	Surgery
Xu [53] 2018 China	1	47 / M	RA	Chronic cough, occasional dizziness for 2 years.	TEE: 25x15 mm mobile mass in RA, attached between CS and IVC.	Surgery: excision.<br>FU: 1 year no recurrence.
Fan 2018 [54] China	1	74 / F	AV	Chest tightness > 10 years.	TTE: 15x10mm mass above non-coronary cusp, no AV interference	Surgery: excision. Mass non-coronary cusp. F 6 MTHs: well.
Ma [55] 2018 U.S.A.	1	60 / FN	MA	CRAO	Ovarian cancer in remission, DM, morbid obesity	Poor surgical candidate, medical: aspirin and warfarin
Nagao [56] 2018 Japan	1	59 / F	MV	ESRD, HD. TTE/TEE: mass MV base, 10x16.9 mm, moving in LV in systole.	DYSA, right arm weakness; brain CT: high-density spot left sylvian fissure.	Surgery: 2 nodules resected from the anterior MV.
Shah [57] 2018 U.S.A.	1	54/M	LV	AC repaired 7 yrs, HYP. TTE: PM calc. mobile mass, 1.2x0.5cm	1 week DYS at rest and PLP.	Surgery

Bhag [58] 2018 India	1	15 days M	LA	TTE: mobile mass from LA, prolapsing MV severe MR	Newborn presented tachypnea 3/6 holosystolic murmur apex	Surgery
Aizawa [59] 2018 Japan	1	38 / F	MV	HD for 31 yrs; ESRD from SLE and nephrotic syndrome	Acute right hemiplegia. TTE/TEE: mobile cord-like mass and MAC	Surgery: MVR and excision
Kasai [60] 2018 Japan	1	75 / M	AV	ESRD, DM, AVS	DYSA; TTE 22x11 mm mobile mass attached to AV post. cusp	Surgery: excision, AVR
Ishida (61) 2019 Japan	1	66 M	AA	ESRD, HD	SYN. TEE: AA mobile flap-like mass 1mm thick, 45x15mm max width.	Surgery: excision
Alizadehasl [62] 2019 Iran	1	43 M	MA MAC	No	ASYM, incidental TTE.	Surgery: excision
Okazaki [63] 2020 Japan	1	67 / M	LV	Gastric cancer, adjuvant CHT, ESRD.	AV endocarditis, fatigue, diarrhea. TTE: mobile 29×18 mm LV apex.	Surgery: excision, AVR. Death 5 months later gastric cancer.
Formelli [64] 2020 Italy	1	79 / F	LA	HYP, moderate obesity, restr. lung disease, polyglobulia; 2 yrs earlier minor stroke	Left hemiparesis, DYSA, left hemineglect; symptoms resolved. TTE/TEE: LA mass 15×18×22 mm	Medical therapy, anticoagulant; surgical risk considered too high
Saku [65] 2020 Japan	1	78 / M	LA	HYP, CAD. Angio CT presented a partially calc mass.	Exertional DYS. TTE and TEE show LA mobile mass 13×16mm	Surgery
Yamanaka [66] 2020 Japan	1	86 / F	MV	Multiple myeloma, CHT since the age of 82.	Incidental. TTE showed a high echoic mass attached to the MV	Surgery: a whitish LAA mass, 4x0.5 cm prolapsing in MV.
Kanemitsu [67] 2020 Japan	2	77 / M<br><br><br>52 / M	MA<br><br><br>MA	HD for 10 yrs, MAC; TTE mass post. MA; CT: 35 mm high-density tumor.<br>HD for 8 yrs, MAC. TEE: 10mm mobile mass PLMV / post. MAC	Incidental, ASYM.<br><br><br>Incidental, ASYM.<br>CT: CAT rapid growth in 5 MTHs.	Surgery: MVR, decalcification (cavitron ultrasonic aspirator), MA remade with pericardium.<br>Surgery: MVR and excision mobile CAT posterior MV
Koyama [68] 2020 Japan	1	83 / F	AV	AVS; CT: mass between NCC/LCC	ASYM AVS	Surgery
Yoshida [69] 2020 Japan	1	62 / M	AA	HD 11 yrs, nephroureterectomy for advanc urothelial cancer, HF.	Incidental. TEE: swinging tumor 3.5x1cm in AA, 5 cm above STJ.	Surgery
Harada [70] 2020 Japan	1	79 FA	AV TV	HD for 16 yrs.	SYN. TTE revealed AV stenosis and tumor on AV and TV, suspected also for vegetation.	Surgery: excision, AVR, TVR, CAB RCA.
Parsaie [71] 2021 Iran	3	47 / F<br>28 / M<br><br>21 / M	RV<br>LA<br><br>RV	MTHAL, transfusions<br>MTHAL, transfusions<br><br>MTHAL, transfusions. CT: dilated PT, chronic PE	NK<br>NK, routine TTE shows LA calc. immobile mass 2.3×0.75 cm<br>DYS, fever, hemoptysis, TTE showed RV 50x18.7 mm mass.	Surgery recommended in all cases, but they were only closely monitored. 5-year FU, patients totally asymptomatic.
Suzue [72] 2021 Japan	1	83 / FA	MV	CAD, HYP, CAB, MAC.	Incidental. TTE 1 month post CAB showed MAC; after 5 MTHs TTE 5x8.2mm mass MV swinging in LVOT; 1 MTH later 5x13 mm.	Surgery: excision
Sano [73] 2021 Japan	1	69 / F	AV MV	ESRD, HYP, DM	ASYM. Incident. TTE: masses AV/MV	Surgery: excision both masses.
Handa [74] 2021 Japan	1	76 / F	MV	HYP, DYSL, osteopor. TTE: calc mass 37×24mm post. MV, AV, MR. CT: calc. mass post. MV.	CI, endovascular thrombectomy; fever, blood culture Gamella sp. Diagnosis: infective CAT.	Surgery: excised CAT, MA with pericardium; MVR AVR. LV rupture. Death 3rd day MOF.
Kumar [75] 2021 India	1	46 / F	LV	ESRD, 2 months earlier, right frontoparietal infarct. Critical LAD/PDA stenosis.	HF. TTE showed LVEF 0.35-0.40 and a 1.9 x 1.7 cm mass in LV, attached to the IVS.	Surgery: excision, 2 CAB.
Nishiguchi [76] 2021 Japan	1	67 / F	MV	HYP, DYSL. 5 MTHs visual impairment. MRI: new CI in left occipital lobe. TTE CAT PLMV; TEE: tumor 14×9.2mm.	Visual field impairment. CT: high-density nodule in MV and left vertebral artery stenosis at 5-6 cervical level. TTE: AS, MAC.	Surgery: excision, MVR, AVR
Fukuda [77] 2021 JapanJ	1	76 / F	LVOT	Mixed connective disease	DYS. Brain MRI: multiple infarctions. EKG: SR. TTE: AS, MAC, 5 mm mobile mass in LVOT.	Surgery: excision, AVR. MAC caseous calcif. too large and not removed. FU 1 year: well.
Mohamed Rafi [78] 2021 India	1	37 / M	RA	Smoker / alcoholic past 9 yrs, Left Hemiparesis. CT brain: acute right fronto-parietal infarct.	TTE: heterogeneous mass in RA. CT chest: densely calcified mass extending into IVC 3.4x1.5 cm.	The patient refused surgery; focus on rehabilitation. MT.
Alok [79] 2022 India	1	42 / M	RV	No comorbidities	SYN and DYS on exertion. TTE: mass RV. MRI: pedunculated mass 15x9 mm RV free wall.	Surgery: excision through the RA. Post-operative recovery was uneventful.
Hematian [80] 2022 Iran	1	4 ms baby M	RA IAS	The baby was born at 28 wks due to placental abruption; in intensive care for respiratory distress.	Cyanosis and cardiac murmur. TTE: RA mass 2.5×0.5 cm with a nonhomogeneous calc, large PDA	Surgery: excision and PDA ligation; mass had multiple attachment to IAS extending to IVC. FU 8 MTHs: well.
Suetani [81] 2022 Japan	1	61 / F	LA	ESRD, HD for 6 yrs. TEE: 3 mobile masses in LA, 2 in post. MA, 3rd mass LPMV.	Incidental, infected leg ulcers. TTE: masses in LA suggestive of endocarditis, severe MR / AS.	Surgery: AVR MVR, masses excision
Kimura [82] 2022 Japan	1	82 / F	MV	Multiple CIs. TTE TEE: 14.9x12.1 mm mass arising PLMV.	Bil hearing loss, 1 MTH before CI dental implant & fever >38°	Surgery
Hachiro [83] 2022 Japan	1	56 / M	MA	ESRD, PD for 3 ys	TTE: MAC, mass attached to post MA. CAT grew 10 mm in 6 months	Surgery: excision. FU at 4 ys no recurrence.
Arimoto [84] 2022 Japan	1	69 / F	LV	ESRD, HD for 10 yrs	PLP, chest tightness. TTE and MRI: mobile mass in LVOT.	Surgery: excision.

Liu [85] 2022 China	1	19 / M	RA TV	ESRD, HPT, HYP. TTE: 30x28 mm RA mass, TV partially obstr.	Resting DYS	Surgery
Çinar [86] 2022 Turkey	1	75 / M	MV	2 yrs earlier acute ischemic stroke, AF diagnosed; TTE MAC.	CI. TTE: mass MV. 3D TEE: mass 11x15mm MV LV side.	Surgery: MVR and tumor excision.
Ushioda [87] 2022 Japan	1	86 / F	MA	HYP. RCA 90% stenosis.	SYN. TTE and TEE: MAC / mobile mass, max diam. 12mm, ant. MA.	Surgery: CAB to RCA and mass excision.
Lee [88] 2022 Korea	1	48 / M	LA	HC, epilepsy; 1 MTH earlier foot fract.. TTE LA mass.	Incidental. Chest X-ray showed a cardiac mass. CT LA 2.8x2 cm.	Surgery
Yamana [89] 2022 Japan	1	60 / M	LV	No. TTE / Angio-CT 73×40 mm LV calc. mass.	Ventricular tachycardia	Surgery: mass removal, cryo-ablation at myocardial border.
Sokolowsky [90] 2022 U.S.A.	1	70 / F	AA 3 cm above AV	HD. CT: no a dissection flap, but AA 2.9x0.9 cm calc. mass, post. wall above AV.	HF. TTE revealed a mobile 3-cm aortic mass; MAC was present.	Surgery: Transverse aortotomy and mass removed with a 3-cm section of AA post. wall.
Endo 2022 [91] U.S.A.	1	71 / F	MV on AML in LVOT	ASYM. Multiple myeloma in remission, DM.	TTE incidental diagnosis, mass LVOT 0,4 cm; MV calc.	Surgery. Mass removal, no MV repair. External PM for few days.
Morisaki [92] 2023 Japan	1	69 / M	MV	PF, EMPH, DM.	DYSA caused by acute CI. TEE: MV mass 22 mm diameter, MAC.	Surgery: CAT removal, MA patch repair, MVR.
Ufuk [93] 2023 Turkey	1	58 / M	LV	HPT, 3 yrs earlier TTE: mass in LV, lost in FU.	Intermittent chest pain, palpitations for 5 days	MT. No surgery for infiltrative extent CAT. FU 6 yrs: well.
Baghaei Tehrani [94] 2023 Iran	1	72 / M	LV	HPT, asthma. ECG: atrial flutter. MR and TR.	DYS/dizziness/fainting 1 month before. TTE: LV mass 56x34 mm.	Surgery: mass excision, MA repair with Teflon, MVR.
Hama-Karim [95] 2023 Iraq	1	14 / F	SVC RA	Homocystinuria. Contrast CT: an intracavitary hyperdense mass in the SVC and RA.	DYS and leg edema. CXR calcif. RA/SVC. TTE: mass rt innomin. vein into RA/SVC.	Surgery: excision not complete. RA mass from SVC to IAS, CS, TV septal leaflet to IVC. Postop reg.
Eizawa [96] 2023 Japan	1	80 / F	MA MV	No. TTE and CT: MAC, diffuse calc MV; 2 mobile masses posterior 19x5mm / 5x2mm.	2-week acute vision loss in left eye due to embolus in branch of retinal artery bifurcation.	Surgery: excision of the 2 tumours. FU at 6 months: no recurrence.
Ghabally (97) 2023 Syria	1	37 / M	RA	Healthy. TTE: RA mass 2.59×2.29 cm; TEE: RA mass 3.32×2.05cm.	Non-exertional chest pain, diaphoresis, nausea, SYN, one episode of severe DYS.	Surgery: excision through right thoracotomy. FU at 1 year: no recurrence at 6 months.
Sakurai [98] 2023 Japan	1	79 / F	MA LV	HD for 8 yrs, HYP, MAC. TTE 1 yr earlier regular. CT calc mass MA / LV linear calcification.	DYS, HF. TTE revealed mass in MA and LVOT, moderate AV regurgitation.	Surgery: AVR and excision. The patient survived 2 ys, no recurrence at TTE.
Odujoko 2023 [99] (U.S.A.)	1	34 / F	MV, LV, RV	DM, UC, ESRD, history mesenteric ischemia	Hospitalized for abdominal pain, died 6 dy later for AMI	Autopsy revealed CAT, the largest lesion was 4.0 cm.
Kim [100] 2024 Korea	1	79 / F	LA IAS	DM, previous colon cancer, normal renal function	ASYM, Chest CT health check-up: 2 cm in LA IAS	Surgery: excision; discharged.
Li [101] 2024 U.S.A.	1	34 / F	LA	Lupus, ESRD, AF.	Confusion, visual changes. CT: strokes in hemispheres/cerebella. TTE: LA mass across MV.	For ESRD and poor general status, removal utilizing the catheter ”AngioVac”
Spencer [102] 2024 U.S.A.	1	74 / F	MA	HYP, DM, venous thrombosis right leg	Left eye vision loss occlus branch retinal artery. TTE: mass 10x11mm MS LV in LVOT; patent FO right/left shunt; MAC	Surgery: excision through aortotomy, stalk attached to MS; FO closure. Post-operative uneventful, FU well 1 month.
Suleiman [103] 2024 Germany	1	65 / F	RA Eustachian valve	HYP, obesity, ESRD and HD, Granulomatosis with polyangiitis (GPA).	Renal transplant evaluation, TEE: RA mass 8x5 mm. Later DYS and chest pain, TEE: mass increased to 10×18 mm EV junction RA/IVC.	Surgery: excision, RA atriotomy closed with patch. FU 5 MTHs: no recurrence, 1 MTH later kidney transplant.
Ivanovic [104] 2024 Serbia	1	73 / M	MA	HYP, congenital kidney aplasia, status post-AMI and Stroke, renal insuff..	Hospitalized for coronary surgery, pre-op TTE showed 15x7 mm mass arising from posterior MA.	Surgery: excision and coronary bypass. Post-op was uneventful.
Chan [105] 2024 Hong Kong	1	57 / MA	MV (PMVL)	No, only smoker and recent onset AF.	Exertional DYS. TTE: MR; TEE: mass 2.2x2.0x1.9cm PLMV extending to MA, chordal rupt.	Surgery: excision, MVR. Post-operative uneventful.
Salehabadi [106] 2024 Iran	1	38 / M	LA, LV, MV, MA, pericardium	[108]	Stroke; CT/MRI: acute infarction basal ganglia. TTE: large echodensity 25×17mm in post. AV groove extending to base post. LV wall and PLMV.	Surgery unsuitable due to extent pericardial/myocardial involvement. MT: anticoagul. FU: 6 months no recurrence, neurologically improved.
Streian [107] 2024 Romania	1	42 / M	LV	CAD, periph. vasculop., HYP, DYS, altered glyc, overweight, previous AMI	Routine TTE, 3-4 cm pedun. Mass, 6 mnths before mass was not present. Genetic decrease fibrinolytic activity	Surgery: excision through transmitral approach: mass 4.5/3.5/3 cm.
Tsushima [108] 2024 Japan	1	58 / M	MV, PML, annular calc.	ESRD, HD for 2ys.	Stroke. TTE: mass PML	Surgery: removal mass.
Hatori [109] 2024 Japan	1	77 F	AV	HYP, DYS, chest pain.	TTE: mobile mass LVOT, AV NCC, MAC. CAD RCA. CT: concomitant lung cancer.	Surgery: mass removal through aortic valve below NCC/RCC, CABG. Pt died 5 mths later for lung cancer.
Paiva [110] 2025 Portugal	1	49 / M	LV	Regular medical history	Exertional DYS. EKG : SR, LBB. TTE : diffuse LV calc. MRI: bulky intramyoc mass; CT calc. mass.	Medical therapy was optimized, referred to heart failure team.
Wilson [111] 2025 U.S.A.	1	76 / F	MV	HYP, DYSL. TTE: moderate MR and 10 mm pedunculated mass on post MA.	Visual impairment, retinal embolism. 2 months after excision, HF due to AT. TTE/TEE: a new highly mobile anterior MA	Surgery: mass excision. Recurrent CAT was treated with medical therapy. Patient improved clinically.

Fu [112] 2025 China	1	46 / M	RA IAS	TTE: 13.2x11.8mm pedunculated mass IAS	ASYM, normal renal function	Surgery: mass excision; FU 1 year: no recurrence.
Dumani [113] 2025 Albania	1	62 / F	MV	TEE: 10mm filamentous formation PLMV, MR, CAD	6 months before TIA, chest pain	Surgery. Excision, P2 removal; 2 CAB. Post-op uneventful.
Present report	2	30 / F<br><br>31 / F	RA SVC<br><br>RA IVC SVC	DYS, SVC syndrome.<br>CT: occl lesions pulm arteries.<br>DYS, ESRD, HD, HYP	Incidental, pregnancy. TEE / MRI 4 cm calc mass RA from IAS to SVC.<br>TEE: mass 34x25mm in RA and inflow IVC, 2 other masses in RA.	Surgery: excision. Post-op regular. FU: 2 yrs well.<br>Surgery: excision performed on beating heart. FU: 1yr well.

Table S1. Clinical issues of cardiac CAT cases published from 1972 to 2025
